# Supplementary material for: Stacking Model‐Based Classifiers for Dealing With Multiple Sets of Noisy Labels
Source: Biom J. 2025 Mar 12;67(2):e70042. doi: 10.1002/bimj.70042 (PMC11898607; doi:10.1002/bimj.70042)
Supplement: Supplementary file 1 — Supporting Information [file BIMJ-67-e70042-s001.zip › density-based-ensemble-model-main/competitors_real_data/netlab-master/netlab-master/help/gtmprob.htm]

Netlab Reference Manual gtmprob


# gtmprob

## Purpose

Probability for data under a GTM.

## Synopsis

```
prob = gtmprob(net, data)
```

## Description

`prob = gtmprob(net, data)` takes a GTM
structure `net`, and computes the probability of each point in the
dataset `data`.

## See Also

`gtm`, `gtmem`, `gtmlmean`, `gtmlmode`, `gtmpost`

---

**Pages:**
Index


---

Copyright (c) Ian T Nabney (1996-9)
